# The Involvement of Hypoxia in the Response of Neuroblastoma Cells to the Exposure of Atorvastatin

**DOI:** 10.3390/cimb45040218

**Published:** 2023-04-11

**Authors:** Ana Salomé Correia, Lara Marques, Nuno Vale

**Affiliations:** 1 OncoPharma Research Group, Center for Health Technology and Services Research (CINTESIS), Rua Doutor Plácido da Costa, 4200-450 Porto, Portugal; 2 Institute of Biomedical Sciences Abel Salazar (ICBAS), University of Porto, Rua de Jorge Viterbo Ferreira, 228, 4050-313 Porto, Portugal; 3 CINTESIS@RISE, Faculty of Medicine, University of Porto, Alameda Professor Hernâni Monteiro, 4200-319 Porto, Portugal; 4 Department of Community Medicine, Information and Health Decision Sciences (MEDCIDS), Faculty of Medicine, University of Porto, Alameda Professor Hernâni Monteiro, 4200-319 Porto, Portugal

**Keywords:** drug repurposing, atorvastatin, neuroblastoma cells, hypoxia-inducible factor-1, echinomycin

## Abstract

Cancer is a set of complex diseases, being one of the leading causes of death worldwide. Despite a lot of research on the molecular pathways and effective treatments, there are still huge gaps. Indeed, the development of new anti-cancer drugs is a complex process. To face this problem, drug repurposing is being increasingly applied. This approach aims to identify new indications for already approved drugs. In this regard, statins (clinically used for reducing cholesterol levels) are reported to induce anti-cancer effects, particularly by inducing apoptosis and altering the tumor microenvironment. Atorvastatin is a type of statin with several potentialities as an anti-cancer agent, supported by several studies. Our study aimed to explore the effect of this drug in SH-SY5Y human neuroblastoma cells. Additionally, we also aimed to understand how this drug acts under hypoxia and the inhibition of hypoxia-inducible factor-1 (HIF-1). For that purpose, we assessed cellular viability/morphology after exposure to different concentrations of atorvastatin, with or without chemically induced hypoxia with chloride cobalt (CoCl_2_) and with or without echinomycin (HIF-1α inhibitor). Our results supported the cytotoxic effects of atorvastatin. Additionally, we also revealed that besides these effects, under hypoxia, this drug induced proliferation of the neuroblastoma cells, supporting the importance of different stimuli and environment on the effect of drugs on cancer cells.

## 1. Introduction

Cancer is one of the leading causes of death worldwide, accounting for approximately 10 million deaths in 2020 [1]. A lot of research efforts are being made to improve therapeutics and better understand the complexity of this set of diseases. Despite this huge research effort, the development of new cancer drugs is a hard, complex, and dispendious process, generating little output. To face this problem, drug repurposing/repositioning is being increasingly studied and applied [2,3].

Drug repurposing is an approach that aims to identify new indications for already approved drugs. This methodology allows lower costs and less time until the drug is approved for the new indication, mainly because information regarding toxicity, drug interactions, and pharmacokinetics is already available [2,4]. Several non-cancer drugs have demonstrated anti-cancer properties, being potential treatment options for cancer patients. Some examples are metformin, disulfiram, thalidomide, and chloroquine [3,5,6,7,8].

Statins, being lipid-lowering agents, are a group of drugs clinically used to reduce cholesterol. These drugs are competitive inhibitors of 3-hydroxy-3-methyl-glutaryl-CoA (HMGCR) reductase, the rate-limiting enzyme in the mevalonate pathway, reducing cholesterol biosynthesis and inducing changes in low-density lipoprotein (LDL) receptor expression [9]. Besides the primary mechanism of action, these drugs are also reported to exert anti-cancer effects, particularly by inducing apoptosis, regulating autophagy, and altering the tumor microenvironment, mainly by inhibition of the mevalonate pathway [10]. Additionally, statins also modify cytoskeleton organization by affecting the membrane localization of Rho GTPases, which is important in the process of metastasis [11].

Nevertheless, being very complex, there are several possible targets in cancer therapy, such as hypoxia (low levels of oxygen). In cancer, this phenomenon is important for progression, angiogenesis, metastasis, and resistance to therapy, which are present in most aggressive tumors. Indeed, hypoxia generates high levels of reactive oxygen species that contribute to cancer cell survival and progression [12].

**Figure 1:**
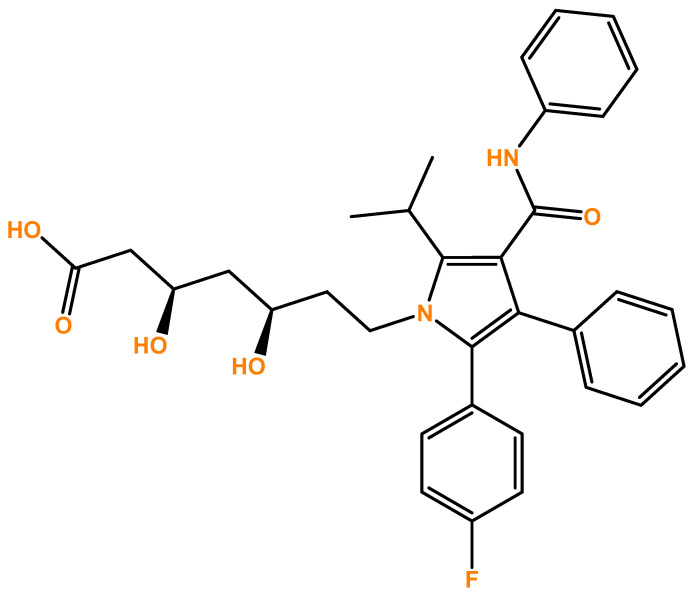
Chemical structure of Atorvastatin.

Atorvastatin (Figure 1) is a type of statin with several potentialities as an anti-cancer agent. Statins were also recently reported to alleviate tumor hypoxia in prostate cancer models by decreasing oxygen consumption [13]. Recent data revealed that this drug, when combined with doxorubicin, led to increased levels of cell death in liver metastasis of breast cancer. Indeed, this drug has potential to be used as an adjuvant and also in conjunction with chemotherapy to reduce the progression of metastatic triple negative breast cancer [14]. Other recent studies also revealed that atorvastatin inhibited the development of prostate cancer, regulating prostate cancer’s cell migration and invasion, and inhibition of the epithelial-mesenchymal transition and matrix metalloproteinases expression [15]. In MDA-MB-231 triple-negative breast cancer cells, this drug also induced caspase-dependent apoptosis and downregulated matrix metalloproteinase-2/9, which is important in the development of metastasis [16]. In these cells, atorvastatin also improved cisplatin sensitivity by modulating cholesteryl ester homeostasis [17]. Recently, this statin was also reported to enhance the efficacy of anti-programmed death-ligand 1 (PD-L1) immune therapy by inhibiting extracellular vesicle PD-L1 [18].

Our study aimed to explore the role of atorvastatin with/without hypoxic stimulus in SH-SY5Y human neuroblastoma cells, a widely used and characterized cell line collected from metastatic tumors, having high proliferative capability. Neuroblastoma is the most common tumor of the sympathetic nervous system, making it important to study. These cells are also reported to exhibit moderate levels of dopamine beta hydroxylase activity [19,20].

For that purpose, we evaluated cellular viability and morphology after exposure to different concentrations of atorvastatin, with or without chemically induced hypoxia with chloride cobalt (CoCl_2_). This compound is widely used as a hypoxia-mimetic agent, blocking hypoxia-inducible factor-1 alpha subunit (HIF-1α) protein degradation under normoxia [21]. Indeed, HIF-1 is one of the most important regulators of the hypoxic response, targeting genes especially related to angiogenesis, cell proliferation/survival, and glucose metabolism, essential to cell survival under hypoxia [22,23]. Thus, we also used echinomycin (an HIF-1α inhibitor) to explore the effect of the inhibition of HIF-1α in SH-SY5Y cells treated with atorvastatin.

To predict the potential interaction between atorvastatin and echinomycin, we also employed an in silico approach using ADMET predictor software. Metabolism is one of the most relevant parameters for the study of drug–drug interactions (DDI) since the mechanisms at the level of metabolizing enzymes are mainly responsible for changes in pharmacokinetics (PK) of concomitantly administered drugs [24]. The cytochrome P450 (CYP) 3A subfamily is recognized as a major contributor to the metabolism of statins. The CYP3A4, in particular, is primarily involved in the metabolism of atorvastatin and produces its two main metabolites, *ortho* and *para*-hydroxyatorvastatin. Thereby, this software tool predicted several drug features, particularly at the metabolism level, in order to determine whether echinomycin interact with atorvastatin [25].

Our results highlighted the potential of atorvastatin as a cytotoxic agent. Additionally, we also revealed that besides atorvastatin’s cytotoxic effects, under hypoxia, this drug induced proliferation of the neuroblastoma cells. Indeed, atorvastatin may act on HIF-1 levels, protecting the cells from the harmful effects of hypoxia and, possibly, inducing cancer cell proliferation under hypoxia. We also verified that both atorvastatin and echinomycin are CYP3A4 inhibitors, and the co-administration of atorvastatin with potent CYP3A4 inhibitors leads to an increase in the plasma concentration of this statin, which may potentiate pharmacological activity. Altogether, our results supported the importance of different stimuli and environments on the effect of drugs on cancer cells.

## 2. Materials and Methods

### 2.1. Materials

Dulbecco’s Modified Eagle’s Medium (DMEM; cat. FG0415) and Fetal Bovine Serum (FBS; cat. S0615) were purchased from Millipore Sigma (Merck KGaA, Darmstadt, Germany). From Sigma-Aldrich (Merck KGaA, Darmstadt, Germany), we obtained penicillin/streptomycin (cat. P4333), neutral red solution (cat. no. N2889), thiazolyl blue tetrazolium bromide (MTT; cat. M5655), atorvastatin (cat. PHR1422), echinomycin (cat. SML0477), and cobalt (II) chloride hexahydrate (cat. 255599).

### 2.2. Cell Treatments

Atorvastatin and Echinomycin were dissolved in DMSO (0.1% in cell culture medium), and CoCl_2_ was dissolved in sterilized water (0.1% in cell culture medium). For Atorvastatin, concentrations ranging 10 nM–20 µM were tested. For CoCl_2_ and Echinomycin, concentrations tested were 0.1 mM and 0.1–5 nM, respectively. All the treatments were tested for 48 h. Vehicles were composed of DMSO 0.1% or sterilized water 1%. For double or triple combinations, they were composed of DMSO 0.2%/sterilized water 2% and DMSO 0.3%/sterilized water 3%, respectively.

### 2.3. Hypoxia Induction

For the induction of hypoxia, we used CoCl_2._ Briefly, we applied 0.1 mM CoCl_2_ for 24 h or 48 h to chemically induce hypoxic conditions to the cells.

### 2.4. Cell Culture

SH-SY5Y neuroblastoma cell line was obtained from American Type Culture Collection (Manassas, VA, USA). Cells were maintained at 37 °C, 5% CO_2_, in DMEM supplemented with 10% FBS and 1% penicillin (1000 U/mL)/streptomycin (10 mg/mL), according to recommendations. The cell seeding was proceeded with a density of 1.0 × 10^5^ cells/mL in 96-well plates (200 μL per well).

### 2.5. Neutral Red Assay

Cell viability was evaluated by neutral red (NR) assay. After 48 h exposure to the different treatments, culture medium was removed, and 100 µL of NR medium (1:100 in culture medium) was added to each well. After that, the cells were incubated for 3 h, protected from the light. Then, cells were washed in PBS, and 150 µL/well of NR destain solution (50% of 96% ethanol, 49% deionized water, 1% glacial acetic acid) was added to the cells. Finally, absorbance values (540 nm) were obtained in the automated microplate reader (Tecan Infinite M200, Zurich, Switzerland).

### 2.6. Thiazolyl Blue Tetrazolium Bromide Assay

Cell viability was evaluated by thiazolyl blue tetrazolium bromide (MTT) assay. After 24 h or 48 h exposure to the different treatments, the culture medium was discarded, and MTT (0.5 mg/mL in PBS) was added to each plate well (100 µL per well). After that, each cell plate was incubated for 3 h at 37 °C, protected from the light. Then, MTT was removed, and DMSO (100 µL/well) was added to the cells. Finally, 570 nm absorbance values were obtained using the automated microplate reader.

### 2.7. Cell Morphology Assessment

SH-SY5Y cells’ morphology was assessed after each treatment (24 h or 48 h) using Leica DMI6000 B Automated Microscope (Leica, Wetzlar, Germany).

### 2.8. Statistical Analyses

Statistical analyses were carried out using the software GraphPad Prism 8 (San Diego, CA, USA). The results were expressed as mean ± SEM of three independent culture preparations. Statistical analyses between the vehicle and treatments were performed with one-way ANOVA followed by Dunnett’s multiple comparisons test. Statistical analyses between treatments were performed with one-way ANOVA followed by Tukey’s multiple comparisons test. Statistical significance when *p* < 0.05.

### 2.9. In Silico ADMET Modeling

The physicochemical and ADMET (absorption, distribution, metabolism, excretion, and toxicity) properties of atorvastatin and echinomycin were estimated by ADMET Predictor v10.4 (SimulationPlus, Lancaster, CA, USA). The chemical structures of both drugs were drawn in MedChem Designer v5.5 (SimulationPlus, Lancaster, CA, USA) and loaded to ADMET Predictor. In this software tool, several ADMET descriptors were accurately predicted, such as lipophilic properties (pKa, logP, logD), solubility, and permeability. Additionally, the PK parameters of 10.0 oral dose (dose recommendation) were assessed using fraction absorbed and bioavailability simulations.

## 3. Results and Discussion

### 3.1. The Effect of Atorvastatin on the Viability and Morphology of SH-SY5Y Cells

**Figure 2:**
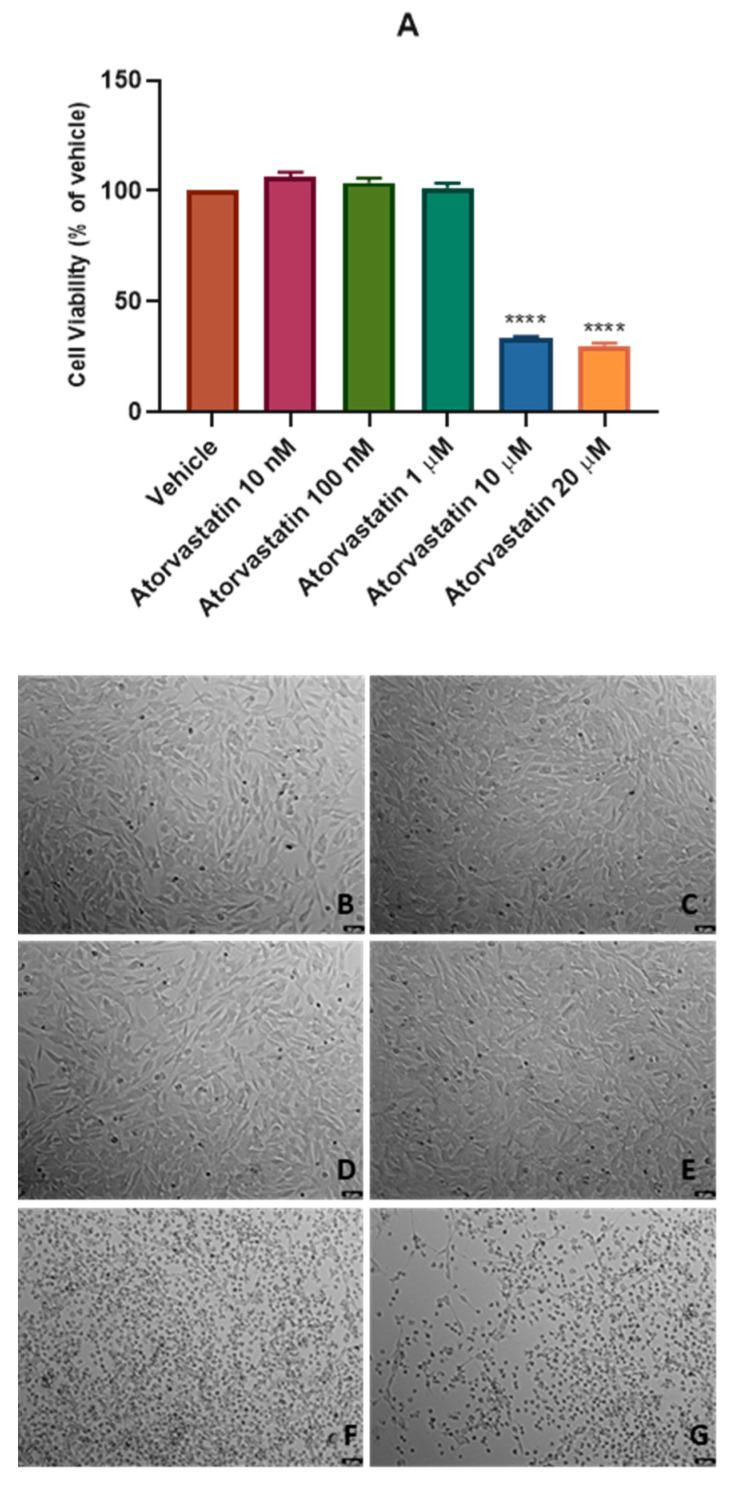
Effect of (**A**) atorvastatin 10 nM–20 µM in SH-SY5Y cellular viability (48 h) obtained by NR assay, and representative images (100 × total magnification) of these cells after incubation with (**B**) vehicle (DMSO 0.1%), (**C**) atorvastatin 10 nM, (**D**) atorvastatin 100 nM, (**E**) atorvastatin 1 µM, (**F**) atorvastatin 10 µM, and (**G**) atorvastatin 20 µM, after 48 h. Cell viability results are expressed as the percentage of the vehicle (100%). Statistical significance at **** *p* < 0.0001 vs. vehicle.

**Figure 3:**
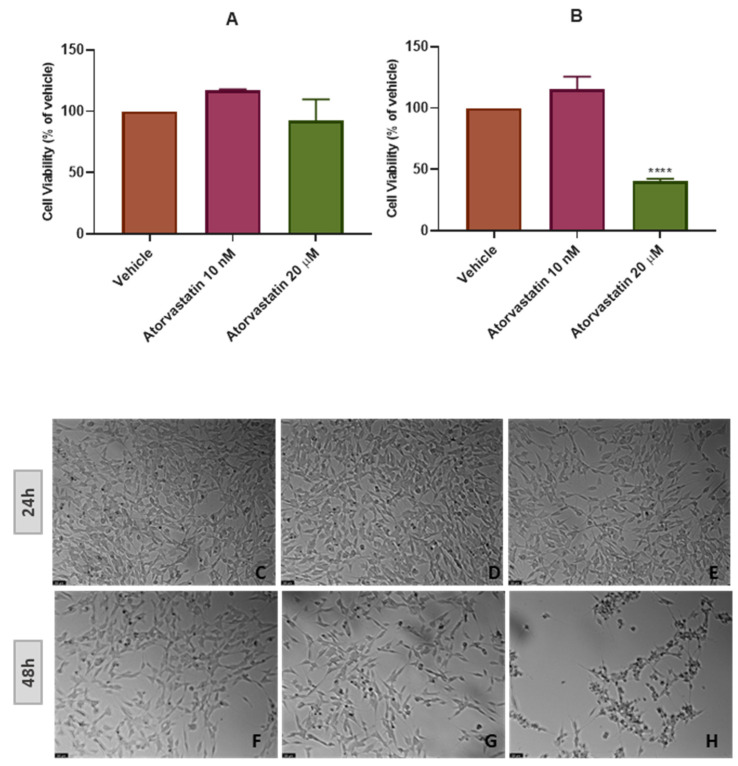
Effect of (**A**) atorvastatin 10 nM and 20 µM in SH-SY5Y cellular viability for 24 h and (**B**) for 48 h obtained by MTT assay, and representative images (100 × total magnification) of these cells after incubation with (**C**,**F**) vehicle (DMSO 0.1%), (**D**,**G**) atorvastatin 10 nM, and (**E**,**H**) atorvastatin 20 µM, after 24 h and 48 h. Cell viability results are expressed as the percentage of the vehicle (100%). Statistical significance at **** *p* < 0.0001 vs. vehicle.

**Figure 4:**
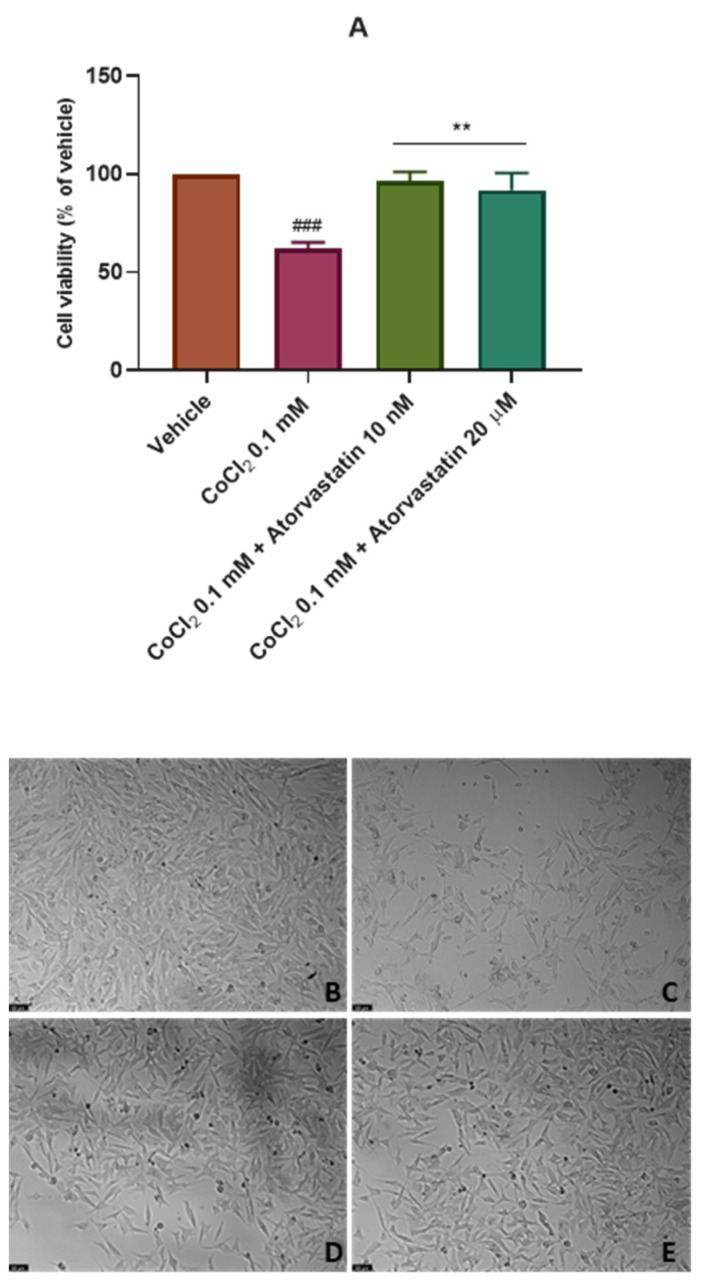
Effect of (**A**) CoCl_2_ 0.1 mM, CoCl_2_ 0.1 mM + atorvastatin 10 nM_,_ and CoCl_2_ 0.1 mM + atorvastatin 20 µM in SH-SY5Y cellular viability (48 h) obtained by MTT assay, and representative images (100 × total magnification) of these cells after incubation with (**B**) vehicle (sterilized water 2%/DMSO 0.2%), (**C**) CoCl_2_ 0.1 mM, (**D**) CoCl_2_ 0.1 mM + atorvastatin 10 nM, and (**E**) CoCl_2_ 0.1 mM + atorvastatin 20 µM, after 48 h. Cell viability results are expressed as the percentage of the vehicle (100%). Statistical significance at ### *p* < 0.001 vs. vehicle and ** *p* < 0.01 vs. CoCl_2_ 0.1 mM.

Aiming to evaluate the effect of different concentrations of atorvastatin in SH-SY5Y cells’ viability and morphology, atorvastatin was added to the cells in concentrations ranging 10 nM-20 μM for 48 h (Figure 2), and in concentrations of only 10 nM and 20 μM for 48 h (Figure 3) and 24 h (Figure 4). Different cell viability methods were employed, namely Neutral Red assay (NR) and Thiazolyl Blue Tetrazolium Bromide Assay (MTT). Briefly, NR assay evaluates lysosomal integrity [26], whereas MTT assesses mitochondrial activity, being the most used cell viability assay [27]. We used NR assay only to determine the effect of the range of the concentrations of atorvastatin to test. After that, we used MTT assay throughout the work, mainly because in our previous experiences, this assay was more reliable and reproducible than NR assay [28]. Nevertheless, it is important to note that both assays were concordant. All the procedures are described in Section 2.

Our results indicate that atorvastatin decreased cellular viability from concentrations of 10 μM, with values of 33.46% vs. vehicle (Figure 2A), for 48 h. Cellular morphology supports these results. Indeed, Figure 2E and F clearly demonstrate cell damage, reflected by rounded morphology, shrinkage/smaller cellular volume, and overall less number of cells, typical features of cell death [29]. These results led us to choose the two extreme concentrations of 10 nM (lower concentration tested) and 20 μM (higher concentration tested) to proceed with our studies. In fact, Figure 3 is concordant with Figure 2, with values of 40.35% (vs. vehicle) for atorvastatin 20 μM and no cytotoxicity with the exposure of atorvastatin 10 nM to the cells for 48 h.

The ideal situation in clinical research is to achieve better results in less time. Thus, we also aimed to test if a shorter period than 48 h (24 h) was suitable to observe SH-SY5Y cells’ response to atorvastatin (Figure 3). It was notorious that this drug did not affect the cellular viability/morphology in this period in the tested concentrations (10 nM and 20 μM). Indeed, viability values of 92.33% (vs. vehicle) were achieved with atorvastatin 20 μM, contrasting with the values obtained with atorvastatin 20 μM for 48 h (40.35% vs. vehicle), demonstrating that 48 h is the suitable period to study the effect of this drug in the viability of SH-SY5Y neuroblastoma cells.

Possibly, by inhibiting the synthesis of cholesterol and its metabolites, atorvastatin decreased cellular viability. Indeed, mevalonate is important in several physiological processes, such as protein prenylation, cell signaling, protein and glycoprotein synthesis, membrane integrity, and cellular respiration, interfering with cellular viability. Several studies suggest that this pathway plays a significant role in the regulation of cellular proliferation and transformation. Nevertheless, besides still not totally elucidated, it has been shown that statins induce apoptosis in sensitive cells directly by inhibition of the HMGCR activity, which is frequently dysregulated in cancer [30].

To date, we have found no studies focusing on the anti-cancer effect of atorvastatin in SH-SY5Y cells. However, studies with other statins supported their effect on human neuroblastoma cells. For example, in a study in these cells, simvastatin stimulated the production of the antiapoptotic protein Bcl-2 via endothelin-1 and nuclear factor of activated thymocytes 3 [31]. Another study with simvastatin also highlighted the apoptotic mechanism of this drug on SH-SY5Y cells by reducing the levels of dolichol, influencing glycosylation process in the endoplasmic reticulum [32].

### 3.2. The Effect of Cobalt Chloride Induced Hypoxia on SH-SY5Y Cellular Viability and Morphology after Atorvastatin Application

**Figure 5:**
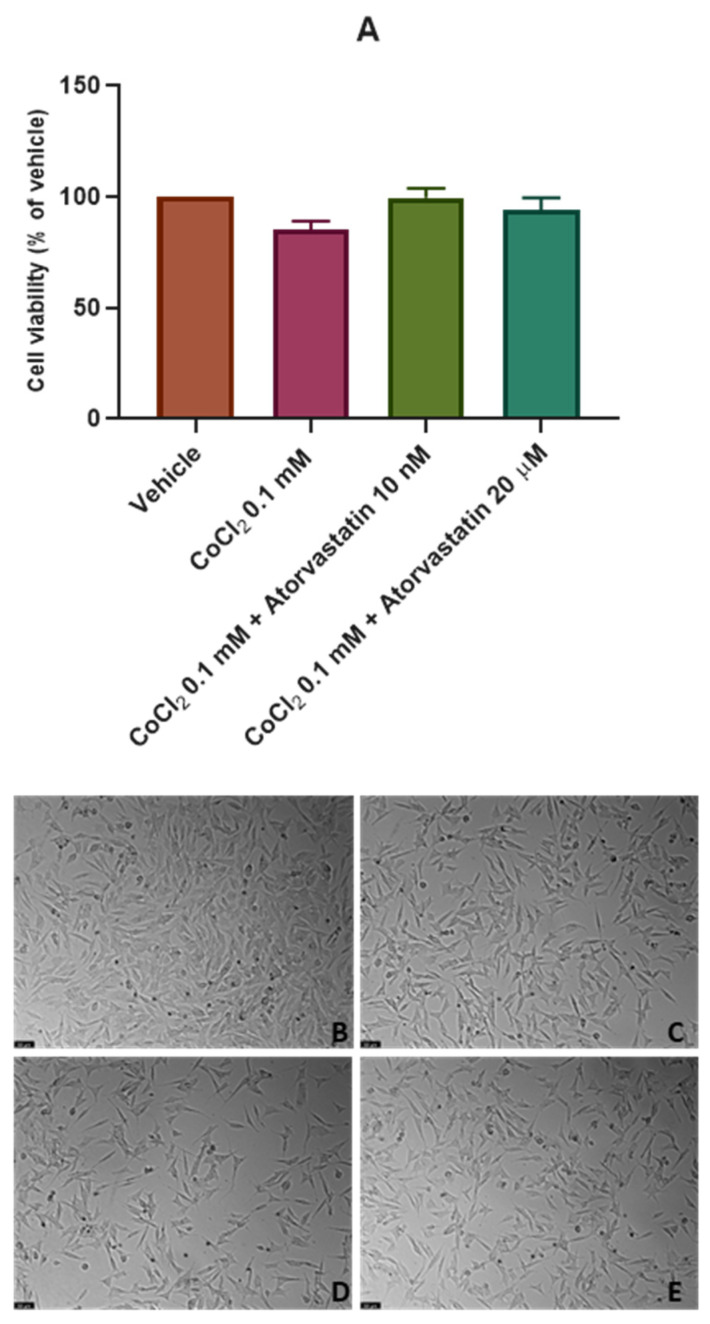
Effect of (**A**) CoCl_2_ 0.1 mM, CoCl_2_ 0.1 mM + atorvastatin 10 nM_,_ and CoCl_2_ 0.1 mM + atorvastatin 20 µM in SH-SY5Y cellular viability (24 h) obtained by MTT assay, and representative images (100 × total magnification) of these cells after incubation with (**B**) vehicle (sterilized water 2%/DMSO 0.2%), (**C**) CoCl_2_ 0.1 mM, (**D**) CoCl_2_ 0.1 mM + atorvastatin 10 nM, and (**E**) CoCl_2_ 0.1 mM + atorvastatin 20 µM, after 24 h. Cell viability results are expressed as the percentage of the vehicle (100%).

After analyzing the effect of atorvastatin on the viability of SH-SY5Y cells, we aimed to test the effect of this drug on these cells after the chemical induction of hypoxia with CoCl_2_. Thus, CoCl_2_ 0.1 mM was combined with 10 nM and 20 μM of atorvastatin for 48 h and 24 h. Cell viability was assessed using MTT (Figure 4A and Figure 5A) and cell morphology was also evaluated (Figure 4B–E and Figure 5B–E). All the procedures are described in Section 2.

These data indicate that for the period of 48 h, atorvastatin attenuated the cell viability decrease after CoCl_2_ application (62.11% vs. vehicle), as observed in Figure 4. Indeed, atorvastatin 10 nM and 20 μM combined with CoCl_2_ led to cellular viability values of 96.67% (vs. vehicle) and, surprisingly, 91.78% (vs. vehicle), respectively. It is important to highlight that the previously observed cytotoxic effect of atorvastatin 20 μM was not observed under chemically induced hypoxia with CoCl_2,_ suggesting that the sensitivity of SH-SY5Y cells to atorvastatin changes after hypoxic conditions.

For a period of 24 h (Figure 5), once again, cells were practically not influenced by CoCl_2_ or atorvastatin. Thus, it was clear that the suitable period for these experiments is 48 h.

Cancer hypoxia is recognized as one of the most important hallmarks of cancer. Indeed, this oxygen deprivation affects cell viability by interfering with gene expression, metabolism, and other signaling pathways [33]. By artificially inducing hypoxia in these cells, viability started to decrease after a period of 48 h. However, the addition of atorvastatin to hypoxic cells reverted this situation. Possibly, atorvastatin induces cell survival mechanisms under a hypoxic environment, such as overexpression of HIF-1, conferring resistance to cells. However, previous studies reported that this drug can inhibit the HIF-1α-PPAR axis on human induced pluripotent stem cells [34] and inhibit serum levels of HIF-1 when combined with routine therapy in rats with acute myocardial infarction [35]. More studies to clarify the role of atorvastatin on HIF-1 expression are needed.

Thus, besides atorvastatin’s cytotoxic effects, under hypoxia, this drug induced proliferation of the cells, supporting the idea that different cellular environments are deeply important for a drug’s response in the context of cancer.

### 3.3. The Effect of the Combination of Echinomycin with Cobalt Chloride on SH-SY5Y Cellular Viability and Morphology after Atorvastatin Application

**Figure 6:**
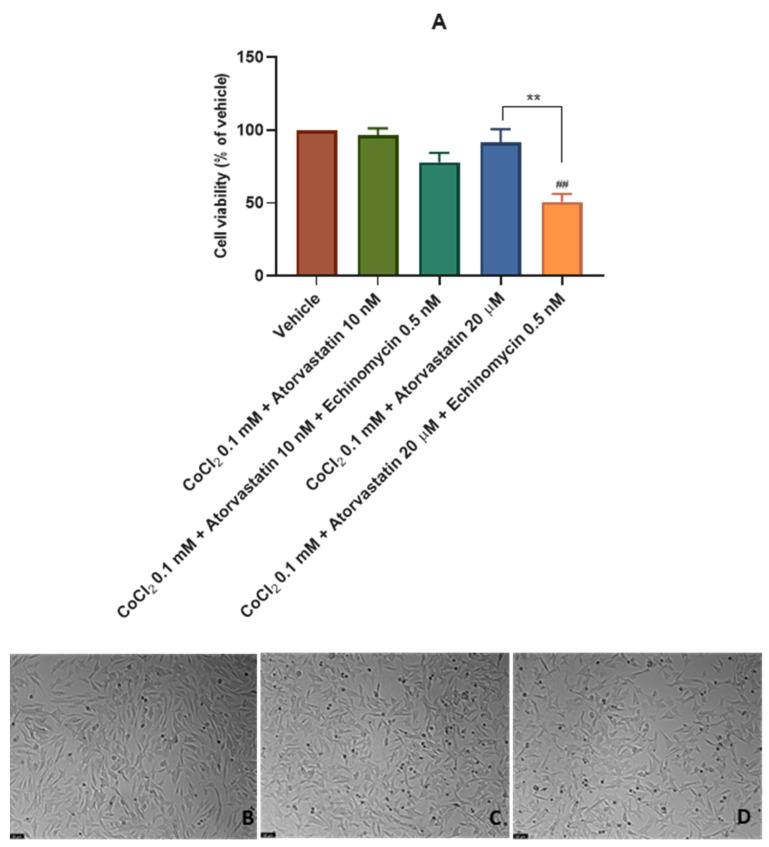
Effect of (**A**) CoCl_2_ 0.1 mM + atorvastatin 10 nM_,_ CoCl_2_ 0.1 mM + atorvastatin 20 µM, CoCl_2_ 0.1 mM + atorvastatin 10 nM + echinomycin 0.5 nM, and CoCl_2_ 0.1 mM + atorvastatin 20 µM + echinomycin 0.5 nM in SH-SY5Y cellular viability (48 h) obtained by MTT assay, and representative images (100 × total magnification) of these cells after incubation with (**B**) vehicle (sterilized water 3%/DMSO 0.3%), (**C**) CoCl_2_ 0.1 mM + atorvastatin 10 nM + echinomycin 0.5, and (**D**) CoCl_2_ 0.1 mM + atorvastatin 20 µM + echinomycin 0.5 nM, after 48 h. Cell viability results are expressed as the percentage of the vehicle (100%). Statistical significance ## *p* < 0.01 vs. vehicle, and ** *p* < 0.01 CoCl_2_ 0.1 mM + atorvastatin 20 µM vs. CoCl_2_ 0.1 mM + atorvastatin 20 µM + echinomycin 0.5 nM.

Finally, 0.5 nM of the HIF-1 inhibitor echinomycin was combined with CoCl_2_ 0.1 mM and 10 nM and 20 μM of atorvastatin for 48 h to explore the effect of atorvastatin on SH-SY5Y cells under chemically induced hypoxia with inhibited activity of HIF-1. Figure S1 demonstrates the individual effect of echinomycin on the viability of SH-SY5Y cells. Cell viability was obtained by MTT (Figure 6A), and cell morphology was also evaluated (Figure 6B–E). All the procedures are described in Section 2. CoCl_2_ 0.1 mM + atorvastatin 10 nM and CoCl_2_ 0.1 mM + atorvastatin 20 μM cell viability values were previously represented in Figure 4. However, they are also represented here in Figure 6A to be easier to compare between conditions (with/without echinomycin).

Our results indicate that atorvastatin 20 μM, when combined with echinomycin under hypoxia induced by CoCl_2_, decreased cellular viability values (50.68% vs. vehicle) compared to the viability values obtained with the combination of atorvastatin 20 μM only with CoCl_2._ (91.78% vs. vehicle). Atorvastatin 10 nM also slightly decreased cellular viability when combined with the HIF-1 inhibitor (77.91% vs. vehicle) compared to the viability values obtained with the combination of atorvastatin 10 nM only with CoCl_2._ (96.67% vs. vehicle).

Altogether, these data indicate that under inhibition of HIF-1 adaptive responses by echinomycin, atorvastatin did not induce cell viability, such as was observed in a hypoxia environment without HIF-1 inhibition. This suggests that atorvastatin may act on HIF-1 levels, protecting the cells from the harmful effects of hypoxia and, possibly, inducing cancer cell proliferation under hypoxic stimuli.

### 3.4. In Silico Validation of the Effect of the Combination of Atorvastatin and Echinomycin

Subsequently, we evaluated the potential interaction between atorvastatin and echinomycin through an in silico approach. ADMET modeling allowed us to obtain a thorough profile of the atorvastatin (Table 1). We then compared ADMET Predictor software-derived values with literature values to assess the prediction accuracy.

The metabolism, at the level of CYP enzymes, was also estimated using this software tool. CYP are a superfamily of enzymes that plays a key role in the metabolism of drugs and other xenobiotics, including environmental pollutants and carcinogens [39].

CYP2C9, CYP2C8, and CYP3A4 are the enzymes involved in the metabolism of atorvastatin, and CYP2C8 and CYP3A4 are known to metabolize echinomycin, as observed in Table 2. Thereby, the presence of two common enzymes (CYP2C8 and CYP3A4) in the metabolism of these two drugs may suggest potential interaction when these are concomitantly administered. CYP enzymes can be inhibited or induced by compounds, resulting in clinically important DDIs. To our knowledge, inhibition and induction mechanisms represent the major contributors to PK DDIs [24].

Co-administration of atorvastatin with potent CYP3A4 inhibitors leads to an increase in the plasma concentration of this statin, which may boost its pharmacological activity [40,41]. Our results demonstrate that echinomycin is a potential CYP3A4 inhibitor (80% of likelihood).

According to our in vitro results, atorvastatin exhibits a cytotoxic effect on SH-SY5Y cells when given alone. When this statin combined with echinomycin under hypoxia induced by CoCl_2_, a decrease in cellular viability was also observed. Our in silico data support these findings. Echinomycin, a CYP3A4 inhibitor, besides the inhibition of HIF-1, may potentiate the cytotoxic effects of atorvastatin on SH-SY5Y cells.

Furthermore, CYP3A4 can also be inhibited by atorvastatin itself. The implications of this inhibition mechanism are not known. In the future, further interaction tests ought to be done to confirm that atorvastatin’s CYP3A4 inhibition does not influence its own kinetics. The influence of CoCl_2_ was also not included in this part of the study due to ADMET Predictor limitations. This software does not incorporate inorganic compounds. Further studies should be conducted to clarify the influence of PK parameters regarding CoCl_2_ on this combination. The probability of additional enzymes and variables that might augment the PK interaction between atorvastatin and echinomycin should be highlighted.

Thus, in silico results also revealed that the co-administration of atorvastatin with potent CYP3A4 inhibitors leads to an increase in the plasma concentration of this statin, which may boost pharmacological activity, increasing the cytotoxic activity of this drug. As predicted by ADMET predictor, echinomycin is a potential CYP3A4 inhibitor, also supporting the in vitro results regarding the combination of echinomycin and atorvastatin.

We believe that more studies about the potential of atorvastatin and other statins under different stimuli/environments are crucial to better understand how these drugs act as anti-cancer agents and how cancer cells may develop resistance to the effects of these drugs. However, this new evidence reported here may have a strong impact on the study of resistance to drugs repurposed for oncology, and also new pharmacological combination studies.

## Figures and Tables

**Table 1:** Estimated physicochemical and pharmacokinetic properties of Atorvastatin from ADMET Predictor. ND not determined.

Properties	Predicated Value	Observed Value	Reference
Molecular Weight (g/mol)	558.65	558.64	[36]
Ionization constant (pKa)	4.71	4.46	[37]
LogP	4.276	6.360	[38]
LogD	1.640	ND	ADMET Predictor
Solubility (mg/mL)	0.185	1.120	[36]
Permeability measure (cm/s × 10^4^)	1.728	ND	ADMET Predictor
Blood-brain barrier permeability	Low likelihood		
Unbound fraction (fu) (%)	5.037		
Blood/plasma ratio	0.667		
Volume of distribution (Vd) (L/kg)	0.317		
Cmax (ng/mL) *	301.630		
Tmax (h) *	1.760		
AUC_inf_ (ng·h/mL) *	1366.220		
T_1/2_ (h) *	1.730		
Total clearance (CL) (L/h) *	6.560		

* These parameters were estimated by simulating the administration of 10.0 mg oral dose (recommended dose) to an individual.

**Table 2:** Metabolism values of atorvastatin and Echinomycin by CYP enzymes, estimated in the ADMET Predictor. ND, not determined.

Drug	CYP Enzymes	Inhibitor	Substrate	Km (μM)	Vmax (nmol/min/nmol Enzyme)	Clint (μL/min/mg HLM Protein)	Sites of Metabolism
Atorvastatin	2C9	Yes (77%)	Yes (45%)	6.096	1.876	22.467	C33, C19, C3, C5, C17, C2
	2C8	ND	Yes (91%)	ND	ND	ND	C18, C19, C33, C1, C17
	3A4	Yes (80%)	Yes (72%)	17.800	77.080	480.670	C19, C33, C1, C17, C18, C28
Echinomycin	2C8	ND	Yes (91%)	ND	ND	ND	S53, C48, C25, C58, S51, C32, C33
	3A4	Yes (80%)	Yes (81%)	0.621	0.291	52.014	S53

## Data Availability

Not applicable.

## Supplementary Materials

The following supporting information can be downloaded at: https://www.mdpi.com/article/10.3390/cimb45040218/s1, Figure S1: Effect of a 48 h incubation of echinomycin 0.1 nM–5 nM on the viability of SH-SY5Y cells determined by MTT assay. The results represent the mean ± SEM of three independent experiments expressed as the percentage of the vehicle (100%). Statistically significant ** p* < 0.05 vs. vehicle.
