# Supplementary material for: The Involvement of Hypoxia in the Response of Neuroblastoma Cells to the Exposure of Atorvastatin
Source: Curr Issues Mol Biol. 2023 Apr 11;45(4):3333–46. doi: 10.3390/cimb45040218 (PMC10137104; doi:10.3390/cimb45040218)
Supplement: Supplementary file 1 [file cimb-45-00218-s001.zip › Supplementary Material Figure S1.pdf]

current issues in molecular biology

MDPI

Supplementary Materials

Evidence of Increased Resistance in Neuroblastoma Cells after Administration of Drug Atorvastatin

Ana Salomé Correia [1,2,3], Lara Marques [1,3] and Nuno Vale [1,3,4,*]

[1]OncoPharma Research Group, Center for Health Technology and Services Research (CINTESIS), Rua Doutor Plácido da Costa, 4200-450 Porto, Portugal

[2]Institute of Biomedical Sciences Abel Salazar (ICBAS), University of Porto, Rua de Jorge Viterbo Ferreira, 228, 4050-313 Porto, Portugal

[3]CINTESIS@RISE, Faculty of Medicine, University of Porto, Alameda Professor Hernâni Monteiro, 4200-319 Porto, Portugal

[4]Department of Community Medicine, Information and Health Decision Sciences (MEDCIDS), Faculty of Medicine, University of Porto, Alameda Professor Hernâni Monteiro, 4200-319 Porto, Portugal

*Correspondence: nunovale@med.up.pt; Tel.: +351-220-426-537

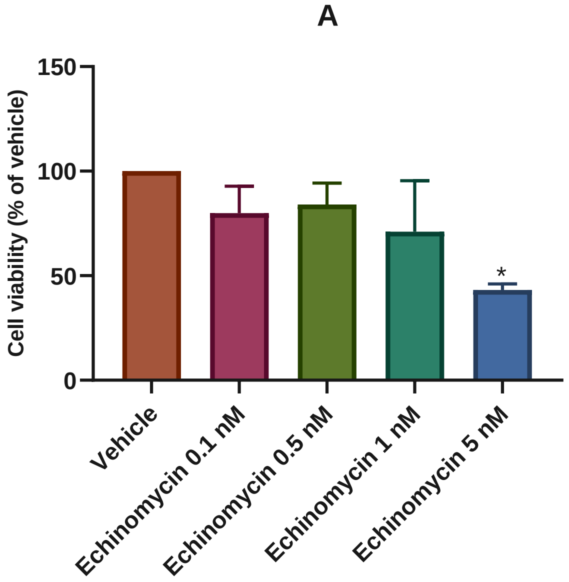

Figure S1. Effect of 48h-incubation of echinomycin 0.1 nM-5 nM, on the viability of SH-SY5Y cells, determined by MTT assay. The results represent the mean ± SEM of three independent experiments, expressed as the percentage of the vehicle (100%). Statistically significant * $p<0.05$ vs. vehicle.
